# The negative effects of short-term extreme thermal events on the seagrass *Posidonia oceanica* are exacerbated by ammonium additions

**DOI:** 10.1371/journal.pone.0222798

**Published:** 2019-09-19

**Authors:** Yaiza Ontoria, Ainhoa Cuesta-Gracia, Juan M. Ruiz, Javier Romero, Marta Pérez

**Affiliations:** 1 Department of Evolutionary Biology, Ecology and Environmental Sciences, University of Barcelona, Barcelona, Spain; 2 Seagrass Ecology Group, Oceanographic Centre of Murcia, Spanish Institute of Oceanography, San Pedro del Pinatar, Murcia, Spain; Università della Calabria, ITALY

## Abstract

Global warming is increasingly affecting our biosphere. However, in addition to global warming, a panoply of local stressors caused by human activities is having a profound impact on our environment. The risk that these local stressors could modify the response of organisms to global warming has attracted interest and fostered research on their combined effect, especially with a view to identifying potential synergies. In coastal areas, where human activities are heavily concentrated, this scenario is particularly worrying, especially for foundation species such as seagrasses. In this study we explore these potential interactions in the seagrass *Posidonia oceanica*. This species is endemic to the Mediterranean Sea. It is well known that the Mediterranean is already experiencing the effects of global warming, especially in the form of heat waves, whose frequency and intensity are expected to increase in the coming decades. Moreover, this species is especially sensitive to stress and plays a key role as a foundation species. The aim of this work is thus to evaluate plant responses (in terms of photosynthetic efficiency and growth) to the combined effects of short-term temperature increases and ammonium additions.To achieve this, we conducted a mesocosm experiment in which plants were exposed to three thermal treatments (20°C, 30°C and 35°C) and three ammonium concentrations (ambient, 30 μM and 120 μM) in a full factorial experiment. We assessed plant performance by measuring chlorophyll fluorescence variables (maximum quantum yield (F_v_/F_m_), effective quantum yield of photosystem II (*ΔF*/*F*_m_’), maximum electron transport rate (ETR_max_) and non-photochemical quenching (NPQ)), shoot growth rate and leaf necrosis incidence. At ambient ammonium concentrations, *P*. *oceanica* tolerates short-term temperature increases up to 30°C. However, at 35°C, the plant loses functionality as indicated by a decrease in photosynthetic performance, an inhibition of plant growth and an increase of the necrosis incidence in leaves. On the other hand, ammonium additions at control temperatures showed only a minor effect on seagrass performance. However, the combined effects of warming and ammonium were much worse than those of each stressor in isolation, given that photosynthetic parameters and, above all, leaf growth were affected. This serves as a warning that the impact of global warming could be even worse than expected (based on temperature-only approaches) in environments that are already subject to eutrophication, especially in persistent seagrass species living in oligotrophic environments.

## Introduction

Climate change represents a major threat to coastal ecosystems worldwide. The urgent need to gain a better understanding of its impact on the performance of organisms and the subsequent cascading effects that cause changes in ecological functions and ecosystem services is a widespread concern [1–3]. Warming is probably the most pervasive effect of global change, and is expected to cause ocean surface temperatures to rise by between 2.6°C and 4.8°C by 2100 [4]. Aside from this progressive warming, most climatic models predict that temperature extremes will increase in frequency and intensity in the coming decades [5–9]. These so-called heat waves increase temperature by several degrees above the historical mean, usually last for days or a few weeks and seem to be especially deleterious for the biota, thereby increasing concern and attracting a great deal of attention in recent years as key drivers of change [7,8,10]. In addition to global warming, a panoply of stressors caused by human activity [11] is already affecting our environment. Thus, warming will impact ecosystems that are heterogeneously affected, to varying degrees, by a range of other stressors, most of them local in origin. The risk that these local stressors could profoundly modify the response of organisms to warming, thereby altering predictions based solely on thermal responses, is gaining attention and in recent years has fostered a growing interest in assessing the combined effects of warming and other stressors [12–14], especially with a view to identifying possible synergies [15,16].

Such a scenario is a particular threat to coastal areas, where human activities are concentrated, thereby generating a wide array of stressors that could potentially interact with warming (continuous or pulsed) and decrease the resilience of the biota. This is especially worrying in the case of foundation species such as corals, gorgonians and seagrasses due to the propagation of the effects, which may extend to other organisms and have ecosystem-wide implications [17–21]. Seagrasses in particular have demonstrated great sensitivity not only to warming [19,22], but also to other stressors of local origin, including eutrophication [23]. Seagrass habitats are considered some of the most valuable coastal ecosystems in terms of the provision of goods and ecological services [24], thus making the assessment of the combined effects of warming and other stressors a major challenge for the scientific community.

Indeed, on the one hand, temperature is widely known to be one of the main ecological factors that determines seagrass performance, survival and distribution limits (see reviews by [25,26]), and the potential effects of temperature rises are subject to an increasing number of studies. It is well known that a moderate temperature rise can be favourable for plant physiology, since it stimulates photosynthesis. However, it also stimulates the respiration rate and, since the latter increases at a faster rate than the former, this can generate a carbon imbalance in plants if it exceeds a certain threshold [22,26–31]. Similarly, it has been demonstrated that photochemical reactions are highly sensitive to thermal stress, which causes damage to the photosystem II (PSII) reaction centres [32,33] that is irreversible beyond a certain threshold (e.g. 37.5°C, *Halophila ovalis* [34]; 40–45°C, *Zostera capricorni*, *Syringodium isoetifolium*, *Cymodocea rotundata*, *Cymodocea serrulata*, *Halodule uninervis*, *Thalassia hemprichii* and *H*. *ovalis* [35]). Negative responses of seagrasses to warming have also been reported at individual and population level, including shoot growth impairment [32,36], an increase in leaf shedding and a reduction in above-ground biomass [33]. In some cases, elevated temperatures have been shown to cause plant mortality [19,22,37] and even alter the geographic limits of seagrass distribution [38,39].

On the other hand, the continuous rise in local nutrient enrichment sources as a consequence of the increasing human population growth and rapid development in coastal areas means that eutrophication is considered a major threat to coastal ecosystems [24,40–42]. Eutrophication can negatively affect seagrasses in particular, either directly or indirectly [43]. The direct effects of nutrient loading, despite the fact that an adequate nutrient supply is fundamental for plant performance [44], include damage caused to seagrasses by excessive inorganic nitrogen (e.g. *Zostera marina;* [45–47]; *Zostera noltii*; [48]). In this sense, the toxicity of high ammonium concentrations has been reported in several studies [48–53], which observed the negative effects of ammonium on several physiological and morphological response variables, including a reduction in primary production and significantly decreased shoot, rhizome and root elongation rates, thus affecting plant survival.

Further research on the isolated effects of each of these two stressors (nutrient loading and warming) on seagrasses is required, but efforts should also be made to assess their combined action, not only to increase knowledge of the expected responses in a realistic multi-stressor scenario, but also to improve the reliability of our predictions about seagrass ecosystem changes in the coming years. In this regard, temperature is already known to exacerbate the negative effects of other stressors such as organic matter-enriched sediments (*Halodule wrightii* and *Thalassia testudinum*, [54]; *Cymodocea nodosa*, [55]) and changes in salinity (*Z*. *marina*; [56]), which act synergistically with thermal stress. Some other works have reported additive effects of temperature and other stressors (e.g. light availability, *Zostera muelleri*; [33]; acidification, *Z*. *noltii*; [32]; and nutrients, *Z*. *marina*; [44]), and, much less commonly, an antagonistic interaction of temperature and a second stressor (e.g. herbicide, *Halophila ovalis*; [57]). All these studies suggest that plant response to the combined impact of temperature and other stressors is largely species-specific and probably depends on the functional traits of the specific plant, but knowledge of this topic with respect to seagrass communities remains scarce and incomplete.

Given the global nature of warming, and the pervasive presence of eutrophication, studying the combined effects of warming and nitrogen loading is crucial to understanding the future of coastal communities dominated by seagrasses, especially in light of the specific plant traits of seagrass foundation species [58]. Although some progress has been made in this area [44,55,59,60], studies that explore this interaction, especially in persistent seagrass species (*sensu* [61]) such as those belonging to the *Posidonia* genus, remain surprisingly scarce.

The Mediterranean endemic species *P*. *oceanica* is an excellent model for exploring the issues described above. On the one hand, *P*. *oceanica* is a paradigm of a persistent species [62,63] and a key foundation species in Mediterranean oligotrophic waters, where it provides critical habitats and other ecosystem services. Due to its high sensitivity to stress and vulnerability to coastal deterioration, *P*. *oceanica* meadows have undergone a substantial decline over the last 50 years [64]. Consequently, it has been one of the main targets of efforts to protect and manage the Mediterranean marine environment in the last 20 years [65]. On the other hand, sea surface temperature in the Mediterranean is increasing at a much faster rate than in the global oceans [6,66] and, at the same time, temperature extremes and heat waves are becoming more common in this region. Moreover, eutrophication is considered a major threat to and stressor for this seagrass, especially near highly populated areas along the Mediterranean coastline, where the first problems of eutrophication were detected as far back as the 1960s [67].

While the effects of eutrophication on this species are relatively well known [68–71], the effects of warming have only recently started being documented [19,27,72–77] and, to the best of our knowledge, there is no information on the potential effects of the interaction between these two stressors.

The aim of this study is thus to explore both the individual and combined effects of warming, by simulating the effects of a short-term extreme temperature event, and eutrophication, through nutrient loading in the form of ammonium, in the persistent seagrass species *P*. *oceanica*. In order to achieve this, we evaluated physiological and individual plant responses to a short-term temperature increase (lasting days) and the interactive effects of ammonium additions. To do so, we conducted an indoor mesocosm experiment in which plants were exposed to three thermal treatments and three levels of ammonium concentration in a full factorial experiment.

## Material and methods

### Plant collection

Divers hand-picked healthy plant fragments of *P*. *oceanica* with at least four interconnected vertical shoots (apical shoots were avoided) in late September 2016 from an eight-metre deep meadow in Cala Montgó (42° 06’ 23” N / 3° 10’ 16” E, NE coast of Spain), where allowances to collect plants fragments for scientific purposes are not required.

Plants were transported in aerated tanks to the laboratory and aerated overnight until the experimental setup the following day. The experiment was performed in the University of Barcelona’s Experimental Fields Service.

### Experimental design and setup

For the experiment, we chose three thermal treatments (20°C, 30°C and 35°C) and three ammonium concentrations: ambient seawater (control), 30 μM (moderate) and 120 μM (high).

**Fig 1:**
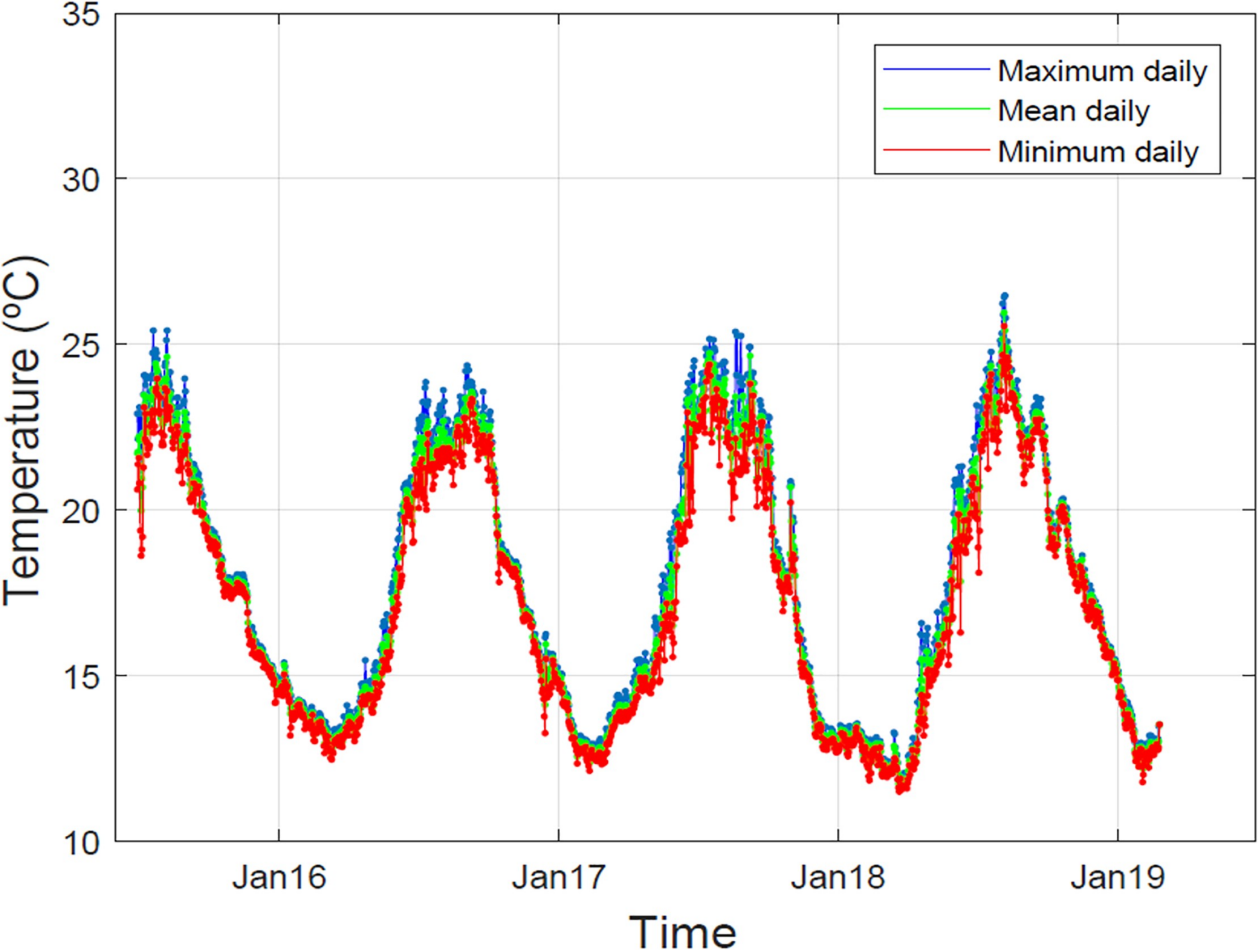
Three-years temperature data series recorded at 5 m deep in Medas Islands (NW Mediterranean Sea).

The temperatures were chosen to represent the following scenarios: 20°C, close to the temperature of the study site at the collection time, according to a temperature data series recorded by continuous *in situ* temperature data loggers (Fig 1), obtained by the authors in Medas Islands (at a depth of 5 m), an area close to the collection site (< 5 km); 30°C, an anomalously high temperature, likely to be reached in the coming years during heat waves (as a reference, > 28°C recorded during recent heat waves by [18,19]), and relatively common in the Eastern Mediterranean basin (Galli et al., 2017) [78]; and 35°C, a temperature during an extreme heat wave that could be reached in the mid-term future (the temperature is predicted to increase by 4–5°C in the western Mediterranean by the end of the 21st century, as per [4,19,79]). With respect to nutrients, the “moderate” value (30 μM) and the “high” value (120 μM) are the lowest and highest values, respectively, observed in sites affected by sewage discharge [80,81] in the Mediterranean Sea, and similar values have been used in previous experimental approaches [44,55].

The plants were incubated in cylindrical and transparent aquaria (12 L capacity, 40 cm height x 20 cm diameter), each with its own independent air pump and filled with 10 L filtered seawater. The plants were incubated in water to avoid possible confounding effects from sediment. Within 24 hours of collection, a single rhizome fragment bearing four interconnected vertical shoots (apical shoots were avoided) was put in each of the 27 aquaria and covered with plastic film to prevent water evaporation. The aquaria were then distributed randomly in three experimental chambers (2 x 1 x 1.5 m, 9 aquaria per chamber), under controlled temperature and light conditions. The chambers were kept at 210–223 μmoles photons m^-2^ s^-1^, above the saturation irradiance of these plants [71,82–84], under a 12h/12h light/dark photoperiod. Light was provided by daylight fluorescent tubes.

The three chambers were maintained at 20°C for four days to allow for plant acclimation. After the acclimation period, the temperature was progressively increased (at a maximum rate of 3°C/day) until it reached 30°C in one chamber and 35°C in the other after 5 days, while the third was kept at 20°C as a control.

After the experimental temperatures were reached, appropriate amounts of NH_4_Cl were added to obtain the ammonium concentration treatments mentioned above. Ammonium was added just once at the beginning of the experiment, to simulate an ammonium pulse.

While thermal treatments were differentiated in three chambers, ammonium treatments were applied to three randomly chosen aquaria in each chamber, which resulted in a complete factorial design with three replicates per experimental condition.

The experiment ended after seven days of exposure to both stressors (temperature and ammonium), when necrosis marks in plants exposed to the highest temperature (35°C) indicated critical damage to the plant.

In order to minimize uncontrolled variability due to small heterogeneities in light and/or temperature, all aquaria were randomly relocated within the chamber every two days. Moreover, each set of nine aquaria was moved from one chamber to another (changing the chamber temperature to maintain the thermal treatments) to ensure that each aquarium spend the same time in each chamber. This was done to discard a potential “chamber effect” and avoid pseudoreplication (Ontoria et al., 2019).

### Water analyses

Nutrients concentration in the water (ammonium, nitrite, nitrate and phosphate) in each aquarium was analysed at the beginning (just after the experimental ammonium additions) and at the end of the experiment, using an FP-2020 Plus Fluorescence Detector, in accordance with standard methodology [85].

### Plant trait response

A number of physiological and individual plant traits were measured at the end of the experiment to determine plant responses. These were maximum quantum yield (F_v_/F_m_), effective quantum yield of PSII (*ΔF*/*F*_m_’), maximum electron transport rate (ETR_max_), non-photochemical quenching (NPQ), incidence of necrosis on the leaves and shoot growth rate.

Chlorophyll fluorescence parameters were determined in three randomly selected shoots from each aquarium using a diving PAM (pulse-amplitude Modulated fluorometer, Walz, Germany). The measurements were obtained from the basal portion of the second youngest leaf to avoid within-shoot variability [86,87]. F_v_/F_m_ was measured by the saturation pulse method after a 10-minute period of dark adaptation. After three hours of illumination, leaves were exposed to increasing photosynthetic photon flux density values (0, 5, 19, 17, 129, 235, 277, 503 and 676 μmol photons m^-2^ s^-1^) at intervals of 10 s to perform rapid light curves (RLCs), which made it possible to obtain *ΔF*/*F*_m_’, ETR and NPQ measurements. *ΔF*/*F*_m_’ and NPQ values extracted from RLCs were those obtained at a similar irradiance to plants that were maintained (210–223 μmoles photons m^-2^ s^-1^), while ETR_max_ corresponded to the maximum ETR value obtained in each curve.

The necrosis incidence was assessed in leaves from three shoots in each experimental condition. Leaves were carefully separated from each shoot and the percentage of necrotic surface (dark brown or black spots covering leaf tissue) relative to the total leaf surface was visually estimated in each leaf and averaged for each aquarium.

Shoot growth was measured using a leaf marking technique [88] adapted to our species [89,90]. On the first day of the experiment, all shoots in each aquarium were marked by punching a hole just above the ligule with a hypodermic needle. At the end of the experiment, the shoots were harvested, the epiphytes carefully removed, and three shoots separated to measure shoot growth. Each shoot was sorted into old and new tissue. Plant material was dried for 48 hours at 60°C and weighed to obtain the dry weight. Shoot growth rate was expressed as the new tissue produced per shoot and day (mg DW shoot^-1^ day^-1^), and then averaged for each aquarium.

### Statistical procedures

The statistical significance of the effects of temperature and ammonium found between treatments was tested using PERMANOVA analyses based on a similarity matrix created from the Euclidean distances between samples. The aquarium was considered as the experimental unit, with a total of n = 3 replicates for each experimental condition. The value for each variable in each replicate is the averaged value for this variable obtained from the three shoots (subsamples) used from each aquarium. Two fixed factors were used to run the analyses: temperature (three levels: 20°C, 30°C and 35°C) and ammonium (ambient water, 30 μM and 120 μM).

Multivariate PERMANOVA was performed for plant response variables and univariate PERMANOVA analyses were subsequently carried out individually for each plant trait. As the PERMANOVA statistical test is produced by permutation, the usual ANOVA normality assumptions [91] were not necessary. Differences between treatments were evaluated using pairwise comparisons, and a Monte Carlo test was carried out to obtain an alternative p-value in order to validate the analysis when the number of permutations was too low (<999, [92]). All analyses were performed using the Primer v6 statistical package [93] in conjunction with the Windows PERMANOVA+ module [92].

## Results

### Nutrient experimental conditions

The initial ammonium concentrations obtained in water ranged from 0.25–0.7 μM, 32–60 μM and 121–132 μM in samples from the control, moderate and high treatments, respectively. At the end of the experiment, ammonium concentrations were very low (less than 1 μM in most treatments, except in two cases: the control (no ammonium added) at high temperature, where some ammonium production took place, and in the high concentration treatment at 35°C, where the final concentration was ca. 70 μM, 60% of the initially supplied (Table 1). Concentrations of other nutrients were in the normal range for the NW Mediterranean waters and did not change significantly during the experiment.

**Table 1:** Ammonium concentrations (in μM, mean ± SEM, *n* = 3) in the water at the beginning (just after ammonium additions) and at the end of the experiment.

Ammonium treatment	Thermal treatment					
	20°C		30°C		35°C	
	NH_4_^+^ (μM)					
	Initial	Final	Initial	Final	Initial	Final
Control	0.25 ± 0.08	0.43 ± 0.18	0.33 ± 0.14	0.27 ± 0.16	0.73 ± 0.27	3.01 ± 1.97
Moderate	40.24 ± 2.18	0.41 ± 0.12	59.87 ± 16.87	0.28 ± 0.23	32.15 ± 1.40	0.85 ± 0.43
High	131.83 ± 2.27	0.15 ± 0.09	123.77 ± 4.47	0.63 ± 0.26	121.27 ± 4.63	72.49 ± 18.83

### Chlorophyll fluorescence parameters

**Fig 2:**
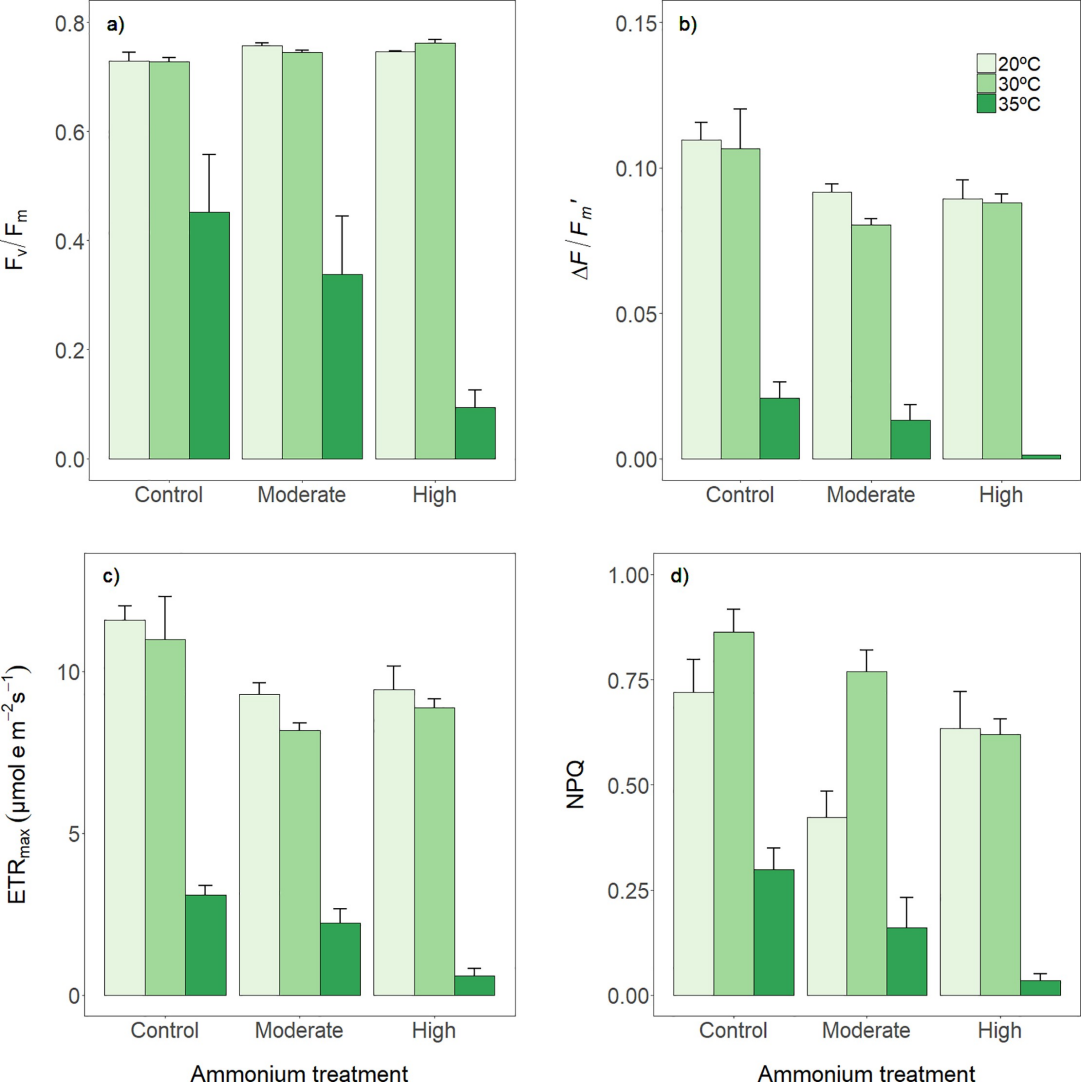
Photochemical responses of *P*. *oceanica* plants to temperature increase and ammonium addition. (a) Maximum quantum yield of dark-adapted leaves (F_v_/F_m_), (b) effective quantum yield of PSII (*ΔF/F’*_*m*_), (c) maximum electron transport rate (ETR_max_), and (d) non-photochemical quenching (NPQ). Each variable was measured (mean ± SE, *n* = 3) at three thermal treatments and at three ammonium concentrations, after 7 days of exposure.

Temperature had a significant effect on all chlorophyll fluorescence parameters measured (Table 2). Maximum and effective quantum yields (F_v_/F_m_ and *ΔF*/*F*_m_’, respectively) and maximum electron transport rate (ETR_max_) showed a similar response pattern, with values at 30°C unaltered and a substantial decrease (38%, 81% and 73%, respectively) at 35°C (in both cases relative to controls at 20°C) (Fig 2A, 2B & 2C). Non-photochemical quenching (NPQ) (Fig 2D) showed slightly higher values at 30°C (up to 17% more) and lower values at 35°C (58%, in both cases relative to controls).

**Table 2:** Results of PERMANOVA (multivariate and univariate analysis) testing for the significance of temperature (20°C, 30°C, and 35°C) and nutrient concentration (C: Ambient; M: Moderate, 30 μM and H: high, 120 μM) effects on plant traits. Bold values indicate significant effects (p<0.05). The results of the pairwise tests are indicated in factors with significant influence.

Variable	Source	df	SS	MS	Pseudo-F	P	Unique perms	Pairwise
	***Main test***							
	Temperature (T)	2	1098.4	549.2	12.857	**0.0004**	9953	
	Ammonium (A)	2	276.8	138.4	3.24	0.0531	9960	
	T X A	4	416.16	104.04	2.4356	0.0741	9950	
	Residual	18	768.9	42.717				
	***Individual test***							
**F**_**v/**_**F**_**m**_								
	Temperature (T)	2	1.21	0.61	76.43	**0.0001**	9935	20 = 30>35
	Ammonium (A)	2	0.05	0.03	3.25	0.0588	9955	
	T X A	4	0.15	0.04	4.75	**0.0073**	9957	35C > 35H
	Residual	18	0.14	0.01	0.00			
***ΔF*/*F***_**m**_’								
	Temperature (T)	2	0.04	0.02	180.52	**0.0001**	9941	20 = 30>35
	Ammonium (A)	2	0.002	0.001	8.99	**0.0019**	9957	C>M = H
	T X A	4	0.0004	0.0001	0.81	0.5396	9946	
	Residual	18	0.0020	0.0001				
**ETR**_**max**_								
	Temperature (T)	2	364.50	182.25	183.78	**0.0001**	9953	20 = 30>35
	Ammonium (A)	2	27.33	13.66	13.78	**0.0005**	9946	C>M = H
	T X A	4	5.06	1.26	1.28	0.3177	9965	
	Residual	18	17.85	0.99				
**NPQ**								
	Temperature (T)	2	1.65	0.83	76.83	**0.0001**	9948	30>20>35
	Ammonium (A)	2	0.21	0.11	9.87	**0.0017**	9952	C>M = H
	T X A	4	0.12	0.03	2.85	0.0582	9952	30C ≥ 30M ≥30H
	Residual	18	0.19	0.01				
**Necrosis**								
	Temperature (T)	2	731.00	365.50	8.76	**0.0019**	9958	20 = 30<35
	Ammonium (A)	2	249.21	124.61	2.99	0.0692	9940	
	T X A	4	410.83	102.71	2.46	0.0725	9960	
	Residual	18	750.71	41.71				
**Growth**								
	Temperature (T)	2	0.0007	0.0004	33.34	**0.0001**	9936	20>30>35
	Ammonium (A)	2	0.0001	0.0001	5.55	**0.0123**	9943	C = M, C = H, M>H
	T X A	4	0.0001	0.00004	3.25	**0.0399**	9956	35C > 35H
	Residual	18	0.0002	0.00001				

Overall, ammonium additions had negative effects in all but one chlorophyll fluorescence parameter (*ΔF*/*F*_m_’, ETR_max_, and NPQ), which decreased by 19%, 19% and 41%, respectively, irrespective of the amount added.

Interestingly, NPQ increased at 30°C in plants submitted to no ammonium addition and moderate ammonium addition but did not at high ammonium concentrations. This is suggestive of a synergistic effect but, given the significance level of the interaction (p = 0.0582), by no means conclusive.

In contrast, the combined effect of temperature and ammonium on decreasing F_v_/F_m_ was clearly synergistic. As mentioned above, warming alone (35°C) depressed F_v_/F_m_ in the absence of ammonium additions, while ammonium additions at the control temperature did not cause any effects (Table 2). However, when ammonium was added and plants were warmed (35°C), F_v_/F_m_ was depressed to 54–87%, relative to controls. At 35°C and under high ammonium concentrations, F_v_/F_m_ was below 0.1, thus indicating critical damage to the photosynthetic apparatus (Fig 2A).

### Leaf necrosis incidence

**Fig 3:**
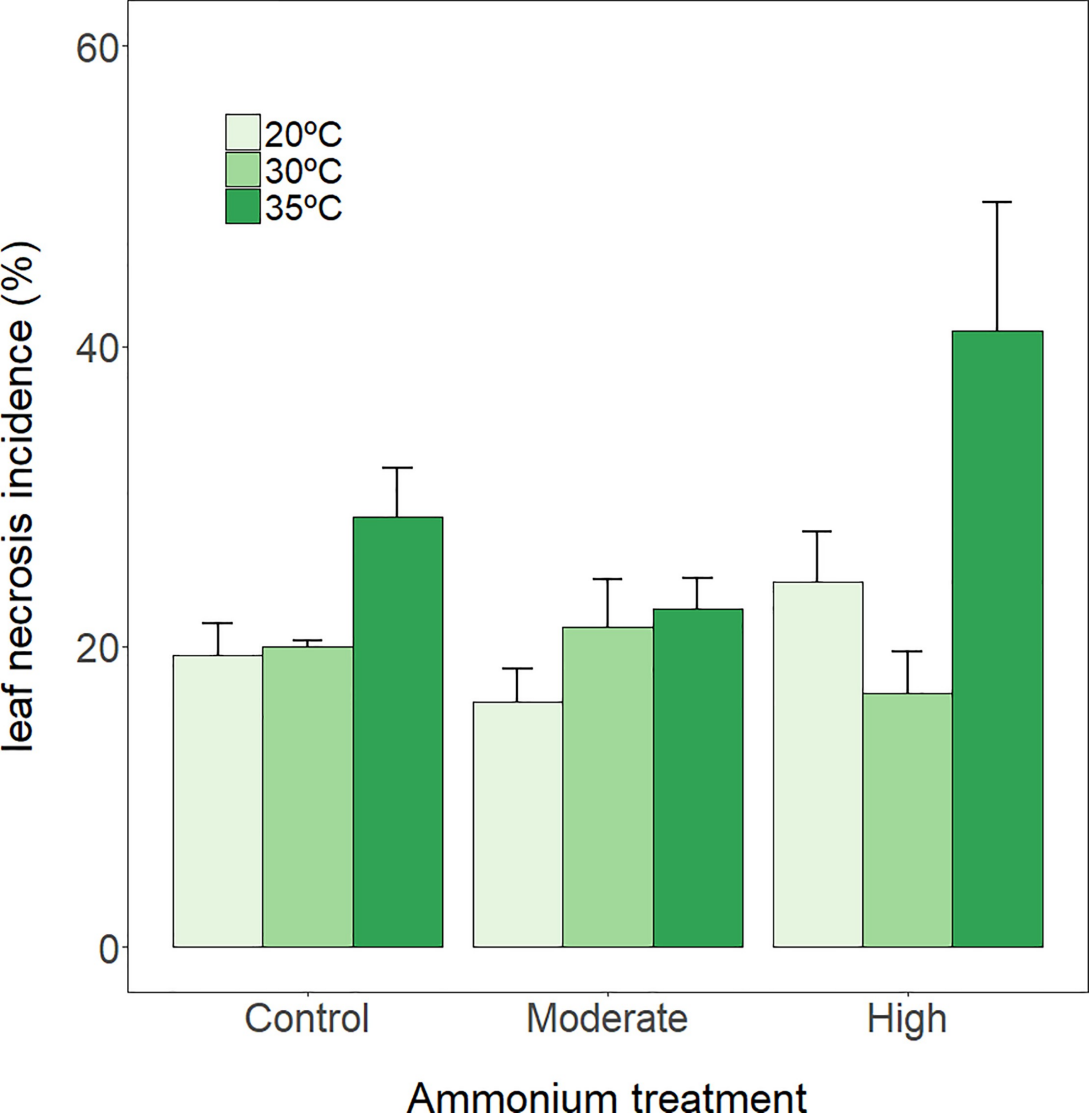
Leaf necrosis incidence. *P*. *oceanica* leaf necrosis incidence (mean ± SE, *n* = 3) at three thermal treatments and at three ammonium concentrations, after 7 days of exposure.

Temperature had a significant effect on leaf necrosis, with an incidence of up to 25% higher at 35°C than at 20°C and 30°C (Fig 3, Table 2). Ammonium addition also appeared to increase necrosis incidence, although the effect was only marginally significant (p = 0.0692), likely due to the high variability of this variable.

### Shoot growth rate

**Fig 4:**
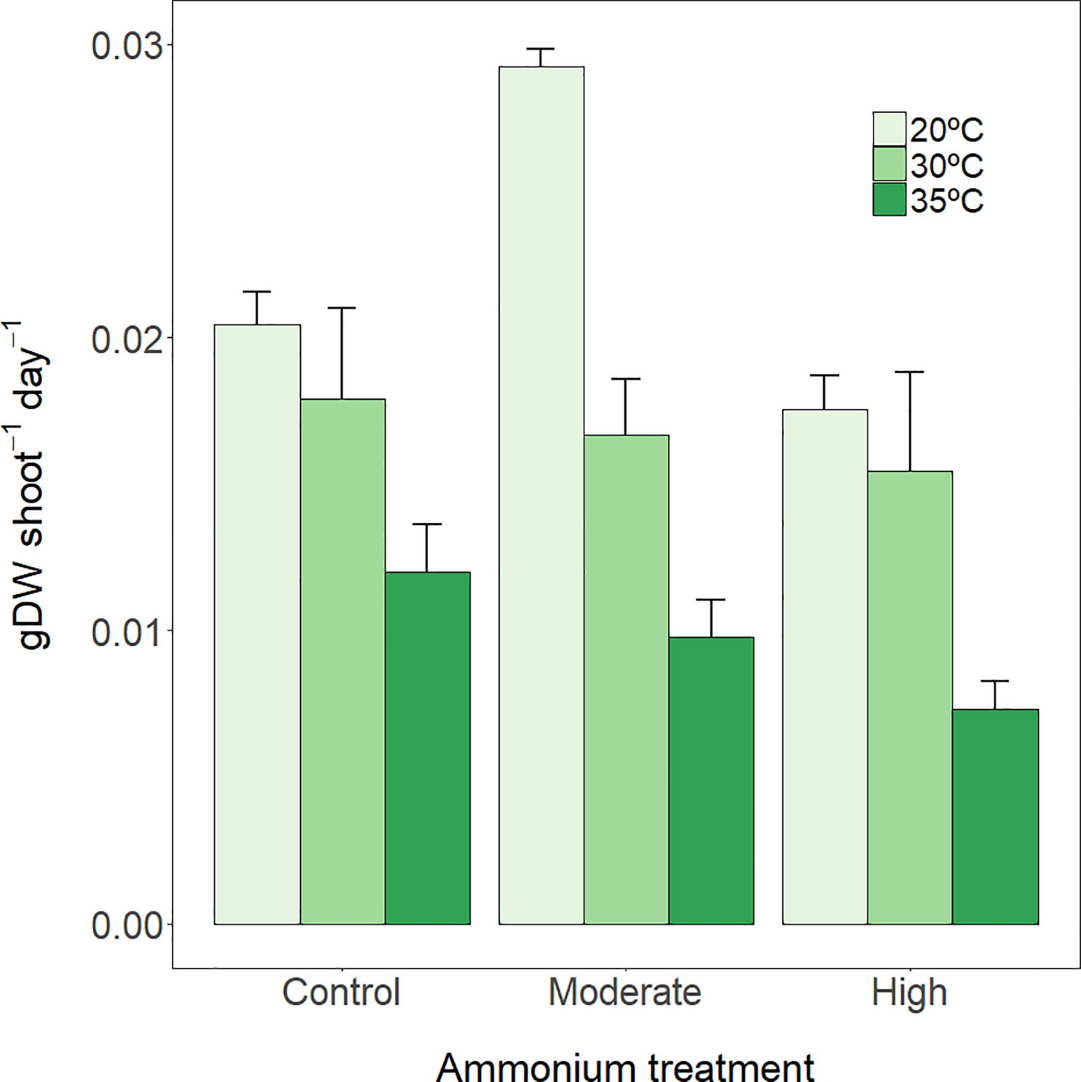
Shoot growth. *P*. *oceanica* shoot growth rate (mean ± SE, *n* = 3) at three thermal treatments and at three ammonium concentrations, after 7 days of exposure.

Both temperature and ammonium had a significant overall effect on plant growth, with a negative effect of temperature and a positive effect (at the moderate concentration only) of ammonium (Fig 4, Table 2). However, these overall effects are misleading, since both stressors showed a clear synergistic interaction (p = 0.0399) that made their combined effect relatively complex. Thus, the positive effect of moderate ammonium concentrations on growth occurred only at the control temperature, while it disappeared at 30°C and became negative at 35°C. Interestingly, the negative effects of extreme temperature (35°C) were considerably higher at the high ammonium concentration (65% growth rate reduction) than at the control ammonium concentration (40%).

## Discussion

While warming has a clear negative effect on most of the variables measured, ammonium additions seem to exert only a moderate impact on plant performance when acting in isolation. However, we detected synergy between both factors in the response of two-three important plant traits, one related to the integrity of the photosynthetic system (maximum quantum yield), the second related to the capacity of the plant to activate photoprotective mechanisms (NPQ, only suggestive, as indicated based on p-value) and the third related to plant production (shoot growth rate), all of which are critical to plant survival. This serves as a warning that the impact of global warming on seagrass meadows already subject to eutrophication could be worse than expected.

A certain amount of interest lies in characterizing the thermal response of foundation species to warming. In the case of *P*. *oceanica*, such studies are relatively scarce (see below). In our case, based on the chlorophyll fluorescence responses and other plant traits, it would seem that *P*. *oceanica* tolerates short-term (i.e. one-week) temperature increases up to 30°C. This tolerance might be partially attributed to the plant’s capacity to activate photoprotective mechanisms (e.g. associated with xanthophyll cycle pigments; [74,94,95]) at this temperature, as suggested by the increasing, albeit not statistically significant, NPQ trend (at 30°C). In addition, neither the necrosis incidence of leaves nor shoot growth were affected by 30°C, in line with the findings of previous studies [76], which would support its thermal tolerance to temperature increases up to 30°C.

By contrast, we observed negative changes in all variables measured at 35°C. Thus, the decrease in F_v_/F_m_ and *ΔF*/*F*_m_’, at 35°C, indicates a severe reduction in the functionality of the photosynthetic apparatus [96]. At the same time, the electron transport chain and, therefore, the electron transport capacity (ETR_max_) were severely affected by this high temperature, which could be attributed to a negative effect on the PSII donor side [97], as reported in previous studies (*Z*. *noltii*, [32]). This suggests that the heat dissipation pathway likely linked to the xanthophyll cycle found at 30°C seems to be inhibited when temperature reaches 35°C, as demonstrated by the drastic reduction in NPQ. This loss of capacity to dissipate the excess thermal energy could have induced damage to the PSII and consequently reduced the photosynthetic capacity of the plants [98]. Impairment of photosynthesis or a likely increase on respiration rates, are probably some of the causes behind the clear reduction in leaf growth that was observed, and certainly triggered other negative effects on plant fitness (reserve accumulation, rhizome growth and probably many others). Finally, the higher leaf necrosis incidence, which is a common plant response to several stressors, including salinity [56,99] and eutrophication [100,101], in plants exposed to 35°C indicates not only a loss of functionality of the photosynthetic systems, but also tissue damage and cell death.

In this regard, based on the thermal sensitivity of this species to high temperatures, as described above [19,77,102], our results and the findings of other studies [27,76,103,104], we suggest a thermal threshold for *P*. *oceanica* of between 30°C and 35°C.

Ammonium additions negatively and moderately affect most of the chlorophyll fluorescence-related variables measured (*ΔF*/*F*_m_’, ETR_max_ and NPQ), independently of temperature (see non-significant interactions in Table 2). No effect of ammonium was detected on F_v_/F_m_ at control or moderately high temperatures (20°C and 30°C). In addition, we observed a positive effect of moderate ammonium addition on shoot growth at the control temperature, consistent with the nutrient-limited condition of this species [69,105]. Therefore, it would seem that the toxicity of ammonium in *P*. *oceanica* at basal temperatures is much lower than in other seagrass species, which are mostly colonizing and opportunistic (*sensu* [61]) species (*Z*. *noltii*; [53]; *Z*. *marina*; [47]). However, the most relevant finding of our experiment was that the negative effects of ammonium additions appear when temperature increases, thus leading to interactive effects between both stressors. Thus, maximum quantum yield (F_v_/F_m_) was clearly affected by ammonium, but only at extreme temperatures (35°C), thereby indicating temperature-dependent ammonium toxicity. This toxicity is likely related to the damage of the photosynthetic machinery which, due to its inability to fix C, hindered the assimilation of ammonium in non-toxic forms [69,106]. In addition, our results suggest that the interaction between both stressors affected the plant’s capacity to activate photoprotective mechanisms, as indicated by a lack of activation of NPQ mechanism at 30°C under high ammonium concentration. Our findings indicate that moderate ammonium additions stimulated shoot growth at control temperature while this stimulation was lost at 30°C and 35°C. Moreover, the thermal effects of extreme temperatures (35°C) were clearly worse at high ammonium concentrations, as growth rates in this treatments combination were 42% lower than those found at 35°C without ammonium addition.

Even though several studies in opportunistic species have revealed that the combined effects of temperature increase and ammonium are not detrimental (*Z*. *marina*, [44,60]; *C*. *nodosa*, [55]), or may even favour plant primary production (*C*. *nodosa*, [59]), our results indicated a negative synergistic effect between both stressors in *P*. *oceanica*, a species considered to be persistent, thus leading to the conclusion that the future impact of warming could be much worse for plants subject to high ammonium loading than for plants living in relatively pristine environments. These findings are consistent with a large number of studies, which have also reported synergistic effects between two simultaneous stressors on seagrasses [39,52,55,107]. However, most of these studies have focused on colonizing and opportunistic seagrass species; further studies are therefore required to shed light on the response of this, and other, persistent seagrass species to simultaneous exposure to two or more stressors.

As highlighted in the introduction, exploring the effects of climate change on coastal ecosystems already threatened by local factors is critical to determining and understanding the future of such ecosystems. Performing factorial experiments, which allow two or more stressors to be combined simultaneously with a view to exploring plant response, could help predict future scenarios.

Although some caution should be exercised when scaling our results up to real-world ecosystems, mainly due to our limited spatial and temporal scales, it is clear that our findings serve as a warning not only about the effects of global warming, but also about the synergies between warming and other local stressors. The predicted rise in the frequency and intensity of heat waves in the Mediterranean Sea [6,66,108] may be tolerated by the plant in the short term, but as duration [27] and/or intensity increase, plant photosynthesis and growth will be curtailed and persistence will likely be compromised. Moreover, other stressors such as eutrophication, especially in persistent seagrass species such as *P*. *oceanica* living in oligotrophic environments, can worsen the negative effects of warming. Consequently, these heightened effects might threat the survival of these important seagrass meadows [108].

Although this research is not fully conclusive, and more extensive experiments, in the field whenever possible, are needed for a proper upscaling to the real world, our results clearly indicate a need to broaden the focus to include the potential interaction with other stressors when attempting to assess the effects of global warming. This is required not only to obtain more accurate, reliable and realistic predictions and therefore aid adaptive management, but also to act against global stressors at local level. In effect, attenuating local stressors may represent one way to alleviate the effects of global warming, or at least ensure they do not worsen.
